# Appearance Time of Methylene Blue in the Aorta: Intra-osseous vs Peripheral Intravenous Route

**DOI:** 10.5812/traumamon.4205

**Published:** 2012-05-26

**Authors:** Mehrdad Hosseinpour, Mohammad Khodaiari

**Affiliations:** 1 Trauma Research Center, Kashan University of Medical Sciences, Kashan, IR Iran

**Keywords:** Methylene Blue, Aorta, Infusion, Intraosseous

## Abstract

**Background:**

The intra-osseous (IO) route serves as an appropriate venous access site if access is needed in an emergency.

**Objectives:**

In this study, we compared the appearance time of methylene blue (MB) in the aorta following IO and peripheral intravenous (IV) routes in a rabbit model to assess a novel idea and compare the speed of IV and IO route of serum delivery into the main circulation.

**Materials and Methods:**

Twenty rabbits were used in our study. They were divided into two groups (odds as G1, n=10, evens as G2, n=10). After laparotomy, the aorta was located and cannulated by a 16 gauge angiocatheter. For IV injection in GII, the marginal vein of either ear was accessed. For IO injection in G1, the medial surface of the proximal extremity of left tibia was used. Once satisfied with positioning, 10 ml of methylene blue solution at a concentration of 10 mg /ml was injected and the time was recorded. The time taken from injection to appearance of MB in the aorta was measured.

**Results:**

All rabbits survived until the end of the experiment. There was no significant difference between the groups regarding the body weight. There was no significant difference between mean time of dye entry into the aorta in either group. It was 9.66 ± 2.51 seconds in G1 and 10.24 ± 1.95 seconds in G2 (P = 0.56).

**Conclusions:**

Our study demonstrated that there was no significant difference between the time taken for MB to reach the central circulation via IO or IV routes.

## 1. Background

Vascular access is best obtained by accessing the upper extremity for the establishment of two large-bore intravenous lines. Multiple different recommendations exist for “failed peripheral access” situations in critical patients. Currently most common recommendations in many emergency wards are IO placement if IV access cannot be achieved within three attempts or 90 seconds (1).The intra-osseous route serves as an appropriate venous access site; however, delivery flow rate of large amounts of crystalloid solutions is limited (2-4).

## 2. Objectives

In this study, we compared the appearance time of methylene blue in the aorta between IO and peripheral IV routes in a rabbit model to introduce a novel idea to compare the speed of peripheral and intraosseous routes of delivery into the main circulation.

## 3. Materials and Methods

Our research ethics committee approved the study. Twenty rabbits were used in our study to compare the entry time of methylene blue solution into the aorta following IO infusion and peripheral IV access routes. White, male, adult New Zealand rabbits (Razi Institute, Tehran, IRAN) weighing 1400-2600 g identified by numbering from l to 20, (tattooed on the internal face of the right ear) were selected. The rabbits were kept in a controlled environment (temperature: 24 - 26˚C, humidity: 55-65 %, fed a commercial pellet diet (Niro –Sahand Co, Tabriz, IRAN) and allowed to freely access tap water until 4 hours before surgery when feeding was discontinued.

They were divided into 2 groups (odds as G1, evens as G2):

Group I (G 1): 10 animals with intraosseous (IO) cannulation.
Group II (G 2):10 animals with peripheral vein (IV) cannulation.

Anesthesia was done with intramuscular premedication of 2 mg/kg body weight acepromazine (30 minutes before anesthesia) and 4 mg/kg body weight xylazine combined with 40 mg/ kg body weight ketamine intramuscularly. The animal was then placed in a horizontal dorsal decubitus position on the surgical table and its paws were fixed to the extremities of the table with thin ropes. Using an electric clipper, abdominal fur was shaved from the region of the abdominis cranialis. Antisepsis of the surgical site was performed with 2 % iodinated alcohol solution. Median laparotomy was performed, starting 1 cm below the processus xiphoideus caudally with 4 cm extension. After exteriorization of intestinal loops, the infrarenal aorta was located and canulated by a 16 gauge angiocatheter. For IV injection, the marginal vein of either ear was accessed. Injection was made by the use of 20 gauge angio-catheter. For IO injection the medial surface of the proximal extremity of the left tibia was shaved and aseptically prepared. In the present study, we used proximal tibia for needle insertion which is the most commonly recommended site for IO access in humans and animals (5).

To access the marrow space through the thick bony cortex, we used a battery–powered hand-held drill. The medullaof the tibia was accessed by a 16 gauge Dieckman IO infusion needle. Entrance into the marrow space was sensed when loss of resistance occurred and the needle stood on its own (6). Once satisfied with positioning, 10 ml of methylene blue solution at a concentration of 10 mg /ml was injected. For controlling rate of administration of the dye, all injections were done by one operator at a rate of 2.5 milliliters/5 seconds. At the beginning of injection, time was recorded by a chronometer. Time was measured from injection to appearance of MB in the aorta (when dye was first seen in aorta catheter by naked eye). Data were checked for normality using Kolmogorov-Smirnov test. Independent student’s t-test was used to compare the appearance time in the groups. P < 0.05 was considered significant. Data were analyzed using SPSS (SPSS Inc, version 12, IL, USA) software.

## 4. Results

The groups were similar in age and sex. There were 10 rabbits in each group. All rabbits survived until the end of the experiment. The mean body weight of rabbits was 1252/2 ± 197.9 gr. There was no significant difference between the groups’ body weight. There was no significant difference between mean time of dye entry to aorta in G1 and G2 (Table 1). It was 9.66 ± 2.51 seconds in G1 and 10.24 ± 1.95 seconds in G2 (P = 0.56)

**Table 1.** Comparison of appearance time (seconds) of methylene blue in the aorta of rabbits injected via peripheral vein (IV) canulation (10 rabbits) and intraosseous (IO) canulation (10 rabbits).

IO Canulation	PV Canulation
10.20	9.59
9.58	8.40
15	7.73
12.02	14.91
7.12	9.46
9.14	10.52
9	11.25
8.14	9.55
10.32	10.37
6.09	10.70

## 5. Discussion

Critical hypovolemia is present in the majority of patients with circulatory shock in the medical–surgical setting presenting as severe trauma, cardiac arrest or respiratory failure and shock. Fluid loading is the first step in treatment and its goal is to optimize left ventricular preload to improve cardiac output. The main aim of treatment is to improve central circulation volume to prevent organ failure. Therefore placement of an intraosseous needle is indicated when vascular access is not feasible in life-threatening situations in babies, infants and children under the age of 6 years. It is also indicated when attempts at venous access fail (three attempts or 90 seconds) or in cases where it is likely to fail and time is crucial. Although principally advocated for use in young children, it has been successfully used in older children. Important complications of this route are tibial fracture especially in neonates, compartment syndrome, osteomyelitis and skin necrosis. When an aseptic technique is used, the incidence of osteomyelitis is less than 1%. Microscopic pulmonary fat and marrow emboli do not seem to be a clinical problem. Provided the correct technique is employed there does not seem to be any long-term effects on bone growth. Factors known to influence the concentration of injected drugs in main circulation include concentration and quantity of drug, volume of injection, rate of injection and site of injection. In 1922, Drinker recognized IO infusion as a viable route for providing vascular access when traditional intravenous methods cannot be accomplished. Despite the wide spread use of IO infusions in adults (7) and multiple studies using several medications detailing kinetics and absorption times for IO administered medication, there are limited studies for direct measurement of time of entry of drugs in the central circulation through IO route. Miller et al. (8) showed that fluid and medication infused via IO lines reach the central circulation within one second equivalent to the speed of an intravenous line. Other authors measured the concentration of various drugs in peripheral vessels such as femoral or antecubital veins to show the efficiency of IO injection. In the Sarrafzadeh-Rezaei study (5), efficiency of induction of anaesthesia by a standard IV route and an IO route was compared. Their results showed that the IO injection of thiopental is a rapid, simple, safe and effective alternative option for induction of general anaesthesia in rabbits. Our study demonstrated that it is possible to inject drugs rapidly into the main circulation intraosseously. It also demonstrated that there were no significant differences between the entry time of methylene blue into the aorta via IO and IV routes. We used the infra-renal aorta in the middle part of animal’s body to equalize the distance of injection sites of the two routes. Although IO is a safe route, it is contraindicated in fractures above the IO insertion site, infection at the insertion site, local vascular compromise and previous orthopedic procedures in the area of insertion, such as total knee replacement.
